# Validation of an Interoperability Framework for Linking mHealth Apps to Electronic Record Systems in Botswana: Expert Survey Study

**DOI:** 10.2196/41225

**Published:** 2023-05-02

**Authors:** Kagiso Ndlovu, Maurice Mars, Richard E Scott

**Affiliations:** 1 Department of Telehealth, School of Nursing & Public Health College of Health Sciences University of KwaZulu-Natal Durban South Africa; 2 Department of Computer Science University of Botswana Gaborone Botswana

**Keywords:** mobile health, mHealth, electronic record systems, eRecord systems, mHealth-eRecord Interoperability Framework, mHeRIF, interoperability architecture, validation, Botswana, mobile phone

## Abstract

**Background:**

Electronic record (eRecord) systems and mobile health (mHealth) apps have documented potential to improve health service delivery, resulting in increased global uptake. However, their interoperability remains a global challenge hindering diagnosis, monitoring of health conditions, and data access irrespective of geographic location. Given the widespread use of mobile devices by patients and health care providers, linking mHealth apps and eRecord systems could result in a comprehensive and seamless data exchange within a health care community. The Botswana National eHealth Strategy recognizes interoperability as an issue and mHealth as a potential solution for some health care needs but is silent on how to make mHealth apps interoperable with existing eRecord systems. A literature review and analysis of existing mHealth interoperability frameworks found none suitable for Botswana. As such, it was critical to conceptualize, design, and develop an mHealth-eRecord Interoperability Framework (mHeRIF) to enhance the interoperability pillar of the Botswana National eHealth Strategy and leverage the full benefits of linking mHealth apps with other health information systems.

**Objective:**

This study aimed to validate the developed mHeRIF and determine whether it requires further refinement before consideration towards enhancing the National eHealth Strategy.

**Methods:**

Published framework validation approaches guided the development of a survey administered to 12 purposively selected local and international eHealth experts. In total, 25% (3/12) of the experts were drawn from Botswana, 25% (3/12) were drawn from low- and middle-income countries in sub-Saharan Africa, 25% (3/12) were drawn from low- and middle-income countries outside Africa, and 25% (3/12) were drawn from high-income countries. Quantitative responses were collated in a Microsoft Excel (Microsoft Corp) spreadsheet for descriptive analysis, and the NVivo software (version 11; QSR International) was used to aid the thematic analysis of the qualitative open-ended questions.

**Results:**

The analysis of responses showed overall support for the content and format of the proposed mHeRIF. However, some experts’ suggestions led to 4 modest revisions of the mHeRIF.

**Conclusions:**

Overall, the experts’ reviews showed that the mHeRIF could contribute to the National eHealth Strategy by guiding the linking of mHealth apps to existing eRecord systems in Botswana. Similarly, the experts validated an architectural model derived from the mHeRIF in support of the first mobile telemedicine initiative considered for national rollout in Botswana. The mHeRIF helps identify key components to consider before and after linking mHealth apps to eRecord systems and is being considered for use as the foundation of such interoperability in Botswana.

## Introduction

### Background

Bidirectional communication between and among eHealth solutions (eg, electronic health-related records and service delivery solutions such as telemedicine) is a desirable goal for access to and portability of functional eHealth. Such functionality is of particular importance for electronic record (eRecord) solutions and mobile health (mHealth) apps as their increased use is inevitable in today’s era of digitally empowered communities. Indeed, eHealth (“the use of Information and Communication Technologies (ICT) for health”) [1] is considered pivotal toward achieving universal health coverage and health-related Sustainable Development Goals [2-4]. mHealth (“mobile wireless technologies for public health”) [5] and eRecord systems are components of eHealth.

In this study, *eRecord systems* refers to electronic health records (EHRs), electronic medical records, and personal health records. Electronic medical records are considered to be internal within an organization and offer real-time patient health records with access to evidence-based decision support tools to aid clinicians in decision-making, whereas EHRs, in contrast, are interorganizational systems hosting longitudinal EHRs over multiple encounters, and finally, personal health records are owned, managed, and shared by individual patients or their legal representatives, resulting in better-informed patients [6,7].

More recently, the COVID-19 pandemic has presented numerous challenges requiring interaction between eHealth components [8]. Moreover, the increasing interest in mHealth solutions has intensified worldwide during the COVID-19 pandemic owing to their ability to promote and support social distancing without compromising the quality or continuity of care [9-11]. This growing application of eHealth is expected to continue after the pandemic [12]. However, despite their documented benefits and continued global uptake [13], the impact of these eHealth components could be increased by ensuring their bidirectional interoperability.

Similar to other *e-words* such as e-business, e-finance, e-government, e-solutions, and e-strategies, the implementation of eHealth systems is never without challenges, both in high-income and low- and middle-income countries (LMICs) [14]. Botswana can be considered an exemplar LMIC with a decentralized network of health care facilities (from national referral hospitals to mobile clinics) across the public and private sectors [15]. eRecord systems in Botswana are not interoperable within or between the public and private sectors, resulting in duplication of effort, manual data sharing, nonstandardized reporting procedures, fragmented care delivery, and unnecessary health care expenditure [15,16]. Botswana’s National eHealth Strategy recognizes mHealth as a potential solution for some health care needs and highlights the need for interoperable eRecord systems at the national level [15]. However, although implied in Figure S1 in Multimedia Appendix 1, the strategy does not directly consider the interoperable linking of these 2 components [15].

mHealth initiatives previously implemented in Botswana supported priority health programs, including cervical cancer screening, oral health, ophthalmology, dermatology, radiology, and tuberculosis, through a coalition of public and private partners [17-22]. Although these initiatives contributed to cost saving and knowledge exchange between health care workers, they were not sustained partly because of their lack of interoperability with existing eRecord systems [16].

Interoperability is the ability of 2 or more systems or components to exchange information and use the information that has been exchanged [23]. It uses standards, interfaces, and protocols to connect systems and services using appropriate software engineering techniques and methodologies and all associated issues [24]. In essence, interoperability is impossible without formal standards and specifications [25]. It can be achieved at various “levels” (technical, syntactic, semantic, organizational, and legal) and provides benefits of real-time information access, improved quality of care, and cost savings [26]. Barriers to achieving interoperability (governance, security and privacy issues, information overload, and liability issues) have been previously identified [16]. Guiding the process are interoperability frameworks offering an agreed upon approach to interoperability for collaborating organizations by specifying common elements such as vocabulary, concepts, principles, policies, guidelines, recommendations, standards, specifications, and practices [27]. Within Botswana, the National eHealth Strategy identifies the need for “establishing a standards and interoperability framework” to inform current and future health care challenges, including early detection of disease and home care monitoring to support personalized care [15].

A previous literature review identified several eHealth interoperability frameworks [28], none of which were found to be entirely suitable or adequate to address the linking of mHealth apps to eRecord systems in Botswana or similar LMICs. A subsequent study developed guidance for Botswana’s National eHealth Strategy for linking mHealth apps to existing eRecord systems by identifying interoperability opportunities and challenges based on local eHealth experts’ opinions and a review of the National eHealth Strategy [16]. This led to the conceptualization, design, and development of the mHealth-eRecord Interoperability Framework (mHeRIF) [29] to extend the scope of Botswana’s National eHealth Strategy by addressing the linking of mHealth apps with eRecord systems (Multimedia Appendix 2).

The proposed mHeRIF highlights the need for governance and regulation of mHealth and eRecord systems. It further shows the need for and role of a national health information exchange (HIE) and is aligned with the established Refined eHealth European Interoperability Framework [27]. These previous studies [16,28] informed key themes, elements, and standards for each interoperability level and sublevel within the mHeRIF. The need to constantly review, audit, and accredit the mHeRIF components was considered essential to addressing emerging challenges and scenarios [29].

The utility of the mHeRIF was demonstrated by developing an interoperability architecture supported by the Open Health Information Exchange (OpenHIE) framework [30] and its reference tool, the Open Health Information Mediator. This was achieved by retrospectively using an exemplar mHealth app requiring linking with existing eRecord systems (the first mobile telemedicine initiative considered for national rollout in Botswana, the Kgonafalo program [31]). Integrating the Healthcare Enterprise workflow profiles were suggested to support various health care scenarios and define shared clinical workflows for the Kgonafalo program [32].

Having demonstrated its utility, it is important to validate the proposed mHeRIF and its OpenHIE-based architecture for Botswana. No specific guidance from the literature addresses the validation of interoperability frameworks linking mHealth apps to eRecord systems. However, literature does exist on the topic of “validation,” and published validation approaches can be considered and either adopted or adapted.

Framework validation approaches have involved psychometric assessment [33], face and content validation [34], and the Delphi method [35,36]. Inglis [37] examined several approaches by going back to the original documents that described the process of development of the frameworks and reported limitations (eg, framework descriptions are usually not sufficiently detailed or clear and often lack any specific mention of validation processes). The study did identify the use of a panel of experts (within a focus group) and suggested a combination of both a literature search and expert opinion as an effective framework validation approach.

### Objectives

In this study, “validation” refers to a process of establishing evidence that confirms that the mHeRIF is “fit for purpose”—capable of consistently guiding the process it is supposed to—and meets the operational needs of its intended users. Given the absence of a specific and accepted validation approach, the recommendation of Inglis [37] was adopted—combining a literature review and expert opinion. Given that a literature review had already guided framework development [28], the aim of this study was to validate the framework through a survey of eHealth experts in Botswana and other countries (high-income countries and LMICs) and refine it as necessary for subsequent use within the implementation of the Botswana National eHealth Strategy.

## Methods

### Overview

The mHeRIF validation process entailed a survey conducted with 12 purposively selected eHealth proponents from academia, industry, and the government. Each was selected based on their experience and expertise in the field within their respective countries or regions. Although the participants’ years in the field were considered, their demonstrable activity within the field (eg, conferences, publications, and reports) was the major consideration. To obtain diverse perspectives, 25% (3/12) of the experts were from Botswana, 25% (3/12) were from LMICs within sub-Saharan Africa (SSA), 25% (3/12) were from LMICs outside SSA, and 25% (3/12) were from high-income countries.

The survey comprised 51 closed-ended questions (26 Likert scale questions and 25 multiple-choice questions [dichotomous or trichotomous]) and 3 open-ended questions. The Likert scale questions related to the design of the proposed mHeRIF. The multiple-choice questions addressed fundamental concepts that guided the framework design and intent, participants’ demographic location, their role in the digital health field, and their years of experience in the field. A total of 3 open-ended questions sought opinions on whether the framework in its current form was suitable to achieve its intent and on areas of possible improvement of the mHeRIF and the associated architecture.

A 4-point Likert scale with a fifth option of “unable to assess” was used for the 26 Likert scale questions (ordinal scale: 4=strongly agree, 3=agree, 2=disagree, and 1=strongly disagree). Closed-ended questions were either dichotomous (*yes* or *no*) or trichotomous (*yes*, *don’t know*, or *no*), and the participants had the option to comment on each of their responses.

The survey was first reviewed by 4 colleagues (nonparticipants in the formal survey) and refined to avoid noted ambiguities. The refined survey was administered on the web using REDCap (Research Electronic Data Capture; Vanderbilt University) forms from March 8, 2022, to June 10, 2022. Survey responses were collated in a Microsoft Excel (Microsoft Corp) spreadsheet, and quantitative data were summarized using descriptive statistics. The NVivo software (version 11; QSR International) was used to aid the thematic analysis of responses to the open-ended questions. The final themes were agreed upon by consensus among all authors and were used to refine the framework.

### Ethics Approval, Informed Consent, and Participation

This study was approved by the Botswana Ministry of Health Research Office (reference HPDME 13/18/1) and the Humanities and Social Sciences Ethics Committee of the University of KwaZulu-Natal, South Africa (reference HSS/0818/015D). All survey participants provided web-based consent. Before participating in the survey, a preliminary personalized email invitation was sent to each potential participant. Those who expressed interest in participating were then sent a formal letter of invitation, a consent form, the developed mHeRIF (with accompanying explanatory notes), and access to a web-based self-administered survey. The explanatory notes (Multimedia Appendix 3 [15,16,27,28,30,32]) described the purpose of the framework and provided definitions of the interoperability concepts as applied to the interoperability architecture and the mHeRIF. This was intended to guide the experts through the framework validation process. The consent forms clearly explained the purpose of the study and provided assurance that the data would be kept safe and deidentified. The participants were informed of their right to refuse to participate or withdraw from the study at any time. No compensation was provided.

## Results

A total of 21 eHealth experts were invited to participate. In total, 13 experts agreed to participate, and 12 (92%) responded to the survey. A total of 17% (2/12) of the experts were in academia, 33% (4/12) were executive leaders, 25% (3/12) were technical officers (eg, analysts or programmers), and the remaining 25% (3/12) were technical managers. Almost all (11/12, 92%) had either 11 to 15 years or >15 years of experience in the field, and 8% (1/12) had 6 to 10 years of experience.

As judged by the median Likert scale scores and most “yes” dichotomous or trichotomous responses, the experts were in agreement with the general format and content of the mHeRIF. All experts (12/12, 100%) “agreed” (*strongly agree* or *agree*) that they were able to understand the mHeRIF structure, interoperability layers, themes, concepts, and their relationships (Table 1). Similarly, all experts (12/12, 100%) agreed that “governance and regulation,” “security, privacy and confidentiality issues,” “mHealth-eRecord standards for interoperability,” “use of terminologies,” “data formats and data models,” and the “need to audit, accredit and align standards for applications and IT infrastructure” were essential components of the mHeRIF (Table 1).

Most eHealth experts (11/12, 92%) further agreed that the mHeRIF offered guidance for linking mHealth solutions to eRecord systems, all the essential interoperability layers were addressed within the mHeRIF, and leveraging open-source eHealth applications such as “Global Goods” (universally available software, services, and content) was an important consideration for LMICs such as Botswana (Table 1). The experts also agreed that “Governance and Regulation” (11/12, 92%), “Human Resource Capacity Building” (12/12, 100%), and legislation (10/12, 83%) were appropriately placed within the mHeRIF. A total of 83% (10/12) of the experts indicated that the mHeRIF could contribute to enhancing a National eHealth Strategy interoperability pillar (Table 1).

Of the 12 experts, 3 (25%) indicated that they would be unable to use the mHeRIF (Table 1). The reasons varied, as noted in optional comments, but each expressed a desire for additional context or information.

Similarly of note is that half (6/12, 50%) of the experts agreed that the mHeRIF satisfactorily addressed fundamental aspects for linking mHealth solutions to eRecord systems (eg, essential communication and network protocols), whereas 17% (2/12) were “unable to assess,” and the remaining 33% (4/12) “disagreed” (Table 1). A total of 42% (5/12) of the experts could not assess whether the mHeRIF satisfactorily aligned with key national policy documents, but this was due to unfamiliarity with these documents. Overall, 25% (3/12) of the experts perceived the mHeRIF as not being suitable to achieve its intent (Table 1). However, participants’ responses to closed-ended Likert scale questions showed an overall median score of 3 (*agree*) for all statements (Table 1; framework design). A score of 4 indicated “strongly agree,” and a response of “U” indicated “unable to assess” for these responses.

A total of 12 dichotomous questions related to “were framework components essential?” The 144 responses are shown in Table 2. Only 8.3% (12/144) of the responses were “No,” whereas 2.8% (4/144) were unanswered; the remaining 88.9% (128/144) of the responses were “Yes” (Table 2). Experts 11 and 12 from high-income countries accounted for most “No” responses (10/12, 83%), with a single expert from a high-income country (expert 12) responding “No” on 8 occasions. The remaining 2 “No” responses were both from experts located in LMICs outside SSA. There was no explanation provided in either case.

**Table 1:** Likert scale responses from eHealth experts on the fundamental concepts that guided the framework development (framework design).

Statement	Likert scale responses^a^																Median score
	Botswana			LMIC^b^ within SSA^c^				LMIC outside SSA				High-income countries					
	Expert 1	Expert 2	Expert 3	Expert 4	Expert 5	Expert 6	Expert 7		Expert 8	Expert 9	Expert 10		Expert 11	Expert 12			
Having read the mHeRIF^d^ description, I am able to understand its structure (interoperability layers, themes, concepts, and their relationships).	4	3	3	3	3	3	3		3	4	3		3	3	3		
The mHeRIF offers guidance to linking mHealth^e^ solutions to eRecord^f^ systems.	3	3	3	3	3	3	3		3	4	3		3	2	3		
I would be able to use the mHeRIF.	4	3	3	3	2	3	2		U^g^	3	3		2	3	3		
The mHeRIF addresses all of the necessary interoperability layers (technical, syntactic, semantic, organizational, and legal).	4	3	3	3	U	3	3		3	4	3		3	3	3		
The mHeRIF promotes leveraging of existing infrastructure such as “on-site” servers and “cloud” technologies to support health care service delivery.	4	3	3	4	3	*2* ^h^	2		U	4	3		U	U	3		
Leveraging open-source eHealth applications such as the “Global Goods” (universally available software, services, and content) is an important consideration for developing countries such as Botswana.	4	4	4	4	4	1	3		4	4	4		3	4	4		
The mHeRIF satisfactorily addresses fundamental aspects to linking mHealth solutions to eRecord systems (eg, essential communication and network protocols).	4	3	3	3	U	*2*	2		U	4	3		2	2	3		
The mHeRIF could contribute to enhancing a National eHealth Strategy interoperability pillar.	4	3	3	4	3	*2*	3		3	4	4		3	U	3		
The mHeRIF satisfactorily aligns with key national policy documents (eg, the “Data Protection Act” and “National ICT policy”) in Botswana.	4	3	3	3	U	*2*	3		U	4	U		U	U	3		
“Governance and regulation” is a relevant component of the mHeRIF.	4	4	4	4	4	4	4		4	4	4		3	3	4		
The “National Health Information Exchange (NHIE)” in the mHeRIF is an essential component to linking mHealth solutions to eRecord systems.	4	4	3	3	3	3	3		4	4	4		4	2	4		
“Human resource capacity building” is a relevant component of the mHeRIF.	4	4	3	4	4	4	3		4	4	4		2	4	4		
Security, privacy, and confidentiality issues are important components within the mHeRIF.	4	4	4	4	4	3	3		4	4	4		3	4	4		
Organizational issues such as “collaboration agreements” and “workflow agreements” are important components within the mHeRIF.	4	3	3	4	U	3	3		4	4	4		3	3	3		
“Usability” of mHealth applications and eRecord systems is an important component of the mHeRIF.	4	3	4	3	U	3	3		4	4	4		3	4	4		
“mHealth-eRecord Workflow Agreements” are important components within the mHeRIF.	4	3	3	3	U	3	3		4	4	4		3	3	3		
Fundamental “mHealth-eRecord” standards for interoperability are essential components within the mHeRIF.	4	3	3	4	4	3	3		4	4	4		4	3	4		
The use of terminologies is an important component to achieve semantic interoperability of mHealth solutions and eRecord systems.	4	4	4	4	4	4	3		4	4	4		3	4	4		
“Data formats” and “Data models” are important components to linking mHealth solutions and eRecord systems.	4	4	3	3	4	3	3		4	4	4		3	3	4		
The need to audit, accredit, and align standards for “Applications” and “IT Infrastructure” within the mHeRIF is essential.	4	3	3	3	4	3	3		4	4	3		3	3	3		
The need to review and align “mHealth-eRecord collaboration agreements” and “mHealth-eRecord workflow agreements” within the mHeRIF is essential.	3	3	3	3	U	3	3		4	4	3		2	U	3		
“Governance and Regulation” is appropriately placed within the mHeRIF.	4	3	3	3	4	3	4		4	4	3		2	3	3		
“Human Resource Capacity Building” is appropriately placed within the mHeRIF.	4	3	3	3	4	3	4		4	4	3		3	3	3		
“Legislation (Security, privacy, and confidentiality)” considerations are appropriately placed within the mHeRIF.	4	3	3	3	2	3	3		4	4	3		3	2	3		
The “unique patient identifier” (UPI) is appropriately placed within the mHeRIF.	4	3	3	3	2	3	3		3	4	4		2	2	3		
Considering the intent of the mHeRIF, which is to guide linking of mHealth apps to eRecord systems in the context of developing countries (using Botswana as the exemplar), and considering the provision that specifics of the content may need to be modified to be context specific: is the framework in its current form suitable to achieve the intent?	3	3	3	3	2	2	3		3	4	3		3	2	3		

^a^A 4-point Likert scale: 4=strongly agree, 3=agree, 2=disagree, and 1=strongly disagree.

^b^LMIC: low- and middle-income country.

^c^SSA: sub-Saharan Africa.

^d^mHeRIF: Mobile Health–Electronic Record Interoperability Framework.

^e^mHealth: mobile health.

^f^eRecord: electronic record.

^g^U: unable to assess.

^h^Italicized Likert scale responses indicate no comments posted for any disagreement (disagree or strongly disagree).

**Table 2:** Validation experts’ responses to multiple-choice questions related to were framework components essential?

Statement	Yes or no responses from local and globally identified eHealth experts											
	Botswana			LMIC^a^ within SSA^b^			LMIC outside SSA			High-income countries		
	Expert 1	Expert 2	Expert 3	Expert 4	Expert 5	Expert 6	Expert 7	Expert 8	Expert 9	Expert 10	Expert 11	Expert 12
The mHeRIF^c^ shows that “Governance and Regulation” is essential?	Y^d^	Y	Y	Y	Y	Y	Y	Y	Y	Y	Y	N^e^
The mHeRIF shows that the “National Health Information Exchange (NHIE)” is an essential component to linking mHealth^f^ solutions to eRecord^g^ systems?	Y	Y	Y	Y	Y	Y	Y	Y	Y	Y	Y	Y
The mHeRIF shows that “Human resource capacity building” is a relevant component?	Y	Y	Y	Y	Y	Y	Y	Y	Y	Y	Y	Y
The mHeRIF clearly shows that security, privacy, and confidentiality issues are essential?	Y	Y	Y	Y	Y	Y	Y	Y	Y	N	Y	N
The mHeRIF shows that organizational issues, such as “collaboration agreements” and “workflow agreements,” are essential?	Y	Y	Y	Y	—^h^	Y	Y	Y	Y	Y	Y	N
The mHeRIF shows that “Usability” of mHealth applications and eRecord systems is an important component?	Y	Y	Y	Y	—	Y	Y	N	Y	N	N	N
The mHeRIF shows that “mHealth-eRecord Workflow Agreements” are essential components?	Y	Y	Y	Y	—	Y	Y	Y	Y	Y	Y	N
The mHeRIF shows that “mHealth-eRecord” standards for interoperability are essential?	Y	Y	Y	Y	Y	Y	Y	Y	Y	Y	Y	N
The mHeRIF shows that use of terminologies (eg, SNOMED-CT^i^ and LOINC^j^) is essential to linking mHealth solutions to eRecord systems?	Y	Y	Y	Y	Y	Y	Y	Y	Y	Y	Y	N
The mHeRIF shows that “Data formats” and “Data models” are essential to linking mHealth solutions and eRecord systems?	Y	Y	Y	Y	Y	Y	Y	Y	Y	Y	Y	N
The mHeRIF shows the need to audit, accredit, and align standards for “Applications” and “IT Infrastructure”?	Y	Y	Y	Y	Y	Y	Y	Y	Y	Y	Y	Y
The mHeRIF shows the need to review and align “mHealth-eRecord collaboration agreements” and “mHealth-eRecord workflow agreements”?	Y	Y	Y	Y	—	Y	N	Y	Y	Y	Y	Y

^a^LMIC: low- and middle-income country.

^b^SSA: sub-Saharan Africa.

^c^mHeRIF: Mobile Health–Electronic Record Interoperability Framework.

^d^Y: yes.

^e^N: no.

^f^mHealth: mobile health.

^g^eRecord: electronic record.

^h^Not available.

^i^SNOMED-CT: Systematized Nomenclature of Medicine–Clinical Terminology.

^j^LOINC: Logical Observation Identifiers Names and Codes.

Of the 3 open-ended questions, 2 (67%) related to the framework (“Is the framework in its current form suitable to achieve the intent?” and “Any suggestions for improvement of the mHeRIF?”) and 1 (33%) related to the architecture (“Any suggestions on how the proposed mHeRIF architecture could be improved to achieve its intent?”). Collectively, the experts provided 15 optional comments to these 3 specific open-ended questions. Considering that the utility of the mHeRIF application was retrospectively demonstrated by developing an architectural model for the Kgonafalo program, none of the experts disagreed with the fundamental concepts that guided the interoperability architecture design and development (Table 3).

In addition, experts sometimes provided optional explanatory comments on their closed-ended responses. Of the 312 opportunities for comments on open-ended questions (26 Likert scale questions × 12 experts), only 98 comments (31.4%) were made. Of the 300 opportunities for comments on dichotomous and trichotomous questions (25 closed-ended questions × 12 experts), 52 (17.3%) were made. Each comment was reviewed by 1 author (KN), and through the process of reflective review, they were categorized into themes. The process was subsequently critically reviewed and revised by a second author (RES). Final agreement was by consensus, with guidance categorized into 7 themes to aid further analysis: “governance and regulation,” “interoperability standards,” “eHealth software and infrastructure,” “unique patient identifier,” “human resource capacity development,” “usability,” and “security, privacy and confidentiality.”

Some of the comments provided informative suggestions for potential framework revision, whereas others provided only general statements that did not offer guidance or require framework revision (eg, “Patient de-duplication generates accurate statistics,” “It is a central factor,” and “I do not have knowledge of these policies”). All comments were reviewed a second time by 2 authors (KN and RES) to parse those that provided potential guidance (Textbox 1) from those that did not, reducing the themes to 6 after the removal of “human resource capacity development” because of the absence of any relevant comments. One comment remained uncategorized: “I believe there should be a nebulous contextual envelope shown explicitly in the diagram” (expert 10; high-income country).

**Figure 1:**
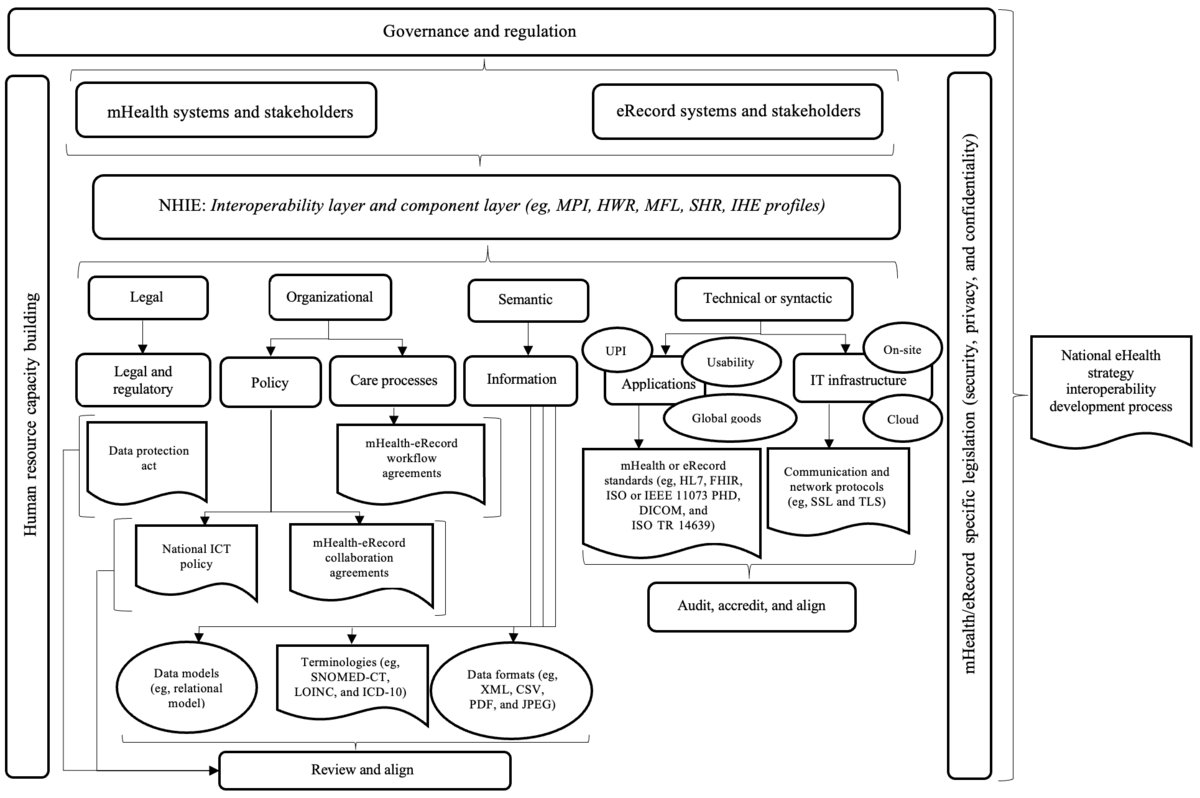
Revised Mobile Health (mHealth) to Electronic Record (eRecord) Systems Interoperability Framework for Botswana presented “primarily” from an enterprise architecture perspective. CSV: comma-separated values; DICOM: Digital Imaging and Communications in Medicine; HL7 FHIR: Health Level 7 Fast Healthcare Interoperability Resources; HWR: Health Worker Registry; ICD-10: International Classification of Diseases, 10th Revision; ICT: information and communications technology; IEEE: Institute of Electrical and Electronics Engineers; IHE: Integrating the Healthcare Enterprise; ISO: International Organization for Standardization; LOINC: Logical Observation Identifiers Names and Codes; MFL: Master Facility List; MPI: master patient index; NHIE: national health information exchange; PHD: Personal Health Data; SHR: Shared Health Record; SNOMED-CT: Systematized Nomenclature of Medicine–Clinical Terminology; SSL: Secure Sockets Layer; TLS: Transport Layer Security; TR: Technical Report; UPI: unique patient identifier.

Some comments led to the revision of the mHeRIF (Figure 1). These are presented in the following paragraphs together with an explanation of what changes were made and where they were made. However, not all suggestions in Textbox 1 were adopted.

**Table 3:** Experts’ opinions on fundamental concepts that guided the interoperability architecture design and intent (N=12).

“Does the interoperability architecture design show evidence of the following:”	Yes or no responses (local and global eHealth experts), n (%)		
	Yes	No	Don’t know
Master patient index (MPI), Health Worker Registry (HWR), Master Facility List (MFL), and Shared Health Records (SHRs) are important registries within the mHeRIF^a^ architecture.	11 (92)	0 (0)	1 (8)
An interoperability layer (OpenHIM^b^) is appropriate within the mHeRIF architecture to support linking mHealth^c^ solutions to eRecord^d^ systems.	11 (92)	0 (0)	1 (8)
The OpenHIE^e^ framework within the mHeRIF architecture is ideal to support linking of mHealth solutions to eRecord systems.	10 (83)	0 (0)	2 (17)
Security, privacy, and confidentiality within the mHeRIF architecture should be prioritized when linking mHealth applications and eRecord systems.	11 (92)	0 (0)	1 (8)
The mHeRIF architecture should support different mobile devices (eg, smartphone, mobile tablet, etc) and platforms (eg, iOS, Microsoft, and Android) as well as future mobile platforms.	11 (92)	0 (0)	1 (8)
The Mobile Device Translation Layer FHIR^f^ Interface supporting implementation of various mobile devices and platforms (eg, iOS, Microsoft, Android) is necessary within the mHeRIF architecture.	10 (83)	0 (0)	2 (17)
Inclusion of the Integrating the Healthcare Enterprise (IHE) profiles within the mHeRIF architecture could enhance interoperability of mHealth solutions and eRecord systems.	9 (75)	0 (0)	3 (25)
The “Mediator” service of the HIE^g^ handling queries and responses between different database systems and resolving complex orchestration of communications between multiple mHealth solutions and eRecord systems is appropriate within the mHeRIF architecture.	10 (83)	0 (0)	2 (17)
Use of telecommunication technologies (eg, SMS, USSD^h^, voice, etc) should form a part of the mHeRIF architecture.	9 (75)	0 (0)	3 (25)
The Case Notification Service (CNS) responsible for sending bidirectional medical case notifications across mHealth and eRecord systems, for example, when a new case is registered using the mHealth solution and resolved through the eRecord system (eg, an EMR^i^) is appropriate within the mHeRIF architecture.	10 (83)	0 (0)	2 (17)
The HL7^j^ FHIR standard in the mHeRIF architecture is ideal for linking mHealth solutions to eRecord systems.	11 (92)	0 (0)	1 (8)
ISO^k^/IEEE^l^ 11073 standards within the mHeRIF architecture are ideal to support interoperability of mHealth solutions and eRecord systems.	7 (58)	0 (0)	5 (42)
The Digital Imaging and Communications in Medicine (DICOM) standard is appropriate within the mHeRIF architecture.	10 (83)	0 (0)	2 (17)

^a^mHeRIF: Mobile Health–Electronic Record Interoperability Framework.

^b^OpenHIM: Open Health Information Mediator.

^c^mHealth: mobile health.

^d^eRecord: electronic record.

^e^OpenHIE: Open Health Information Exchange.

^f^FHIR: Fast Healthcare Interoperability Resources.

^g^HIE: health information exchange.

^h^USSD: Unstructured Supplementary Service Data.

^i^EMR: electronic medical record.

^j^HL7: Health Level 7.

^k^ISO: International Organization for Standardization.

^l^IEEE: Institute of Electrical and Electronics Engineers.

**Validation experts’ comments providing informative suggestions for potential framework revision.**

Governance and regulation

- “Issues of investment should be covered to detail how funding for the projects will be sustained” (Expert 1; Botswana).
- “An investment case through donor funding or PPP arrangements could be suggested for low income countries” (Expert 1; Botswana).
- “It places it at the top and all encompassing from left to right—Author may wish to make it the largest rectangle containing all of the other boxes inside (ala COBIT 2019)” (Expert 7; low- and middle-income country [LMIC] outside sub-Saharan Africa [SSA]).

Interoperability standards

- “Yes for now, but the mHeRIF design should accommodate for a standard component not necessarily tightly coupled to HL7 FHIR” (Expert 4; LMIC within SSA).
- “In principle, I would say yes, however this is not my area of expertise. I have a feeling that depending on the architectural approach, it is not necessarily the role of the exchange to support a wide range of third-party systems. Rather, I believe an approach is for the exchange to expose APIs and publish communication standards/protocols using open standards and then it is the responsibility of the third-party systems to do the work to be able to communicate with the exchange. You may want to check this to make sure I’m not talking nonsense” (Expert 5; LMIC within SSA).
- “I don’t see ‘IHE Profiles’ mentioned anywhere in the figure. Might be good to add it” (Expert 5; LMIC within SSA).
- “The author is also referred to ISO TR 14639 as another target state for eHealth architecture” (Expert 7; LMIC outside SSA).

eHealth software and infrastructure

- “Consider just using the more encompassing and standard term ‘digital health’ and then differentiate their delivery methods and tool such as online/offline (including on-premise/cloud), hardware device such as mobile devices, etc” (Expert 4; LMIC within SSA).
- Telecommunication technologies such as SMS text messaging, Unstructured Supplementary Service Data, and voice “...could be independently mapped by an outer layer in the mobile device/interface” (Expert 10; high-income country).

Security, privacy, and confidentiality

- “I like the place that ‘Security, privacy and confidentiality’ are shown, and I believe these are extremely important. I would not put include ‘Legislation’ where it is. The legislation is already covered under the regulation part of ‘Governance and Regulation.’ Also the role of Security, privacy and confidentiality here are not to engage with or produce the legislation but rather to put practical, technical measures in place that fulfil the requirements of the legislation that is already mentioned in the heading ‘Governance and Regulation’” (Expert 5; LMIC within SSA).

Usability

- “I suggest you align the position and design of the parts of figure 1 and figure 2. This will make the logic easier to follow. For example, security is on the right in Fig 1 and on the left in Fig 2. Audit also moves as do the HIE components” (Expert 5; LMIC within SSA).
- “The framework needs to be further simplified” (Expert 6; LMIC within SSA).
- “There are other aspects such as AI [Artificial Intelligence] and HCI [Human-Computer Interaction] not explicitly provided for” (Expert 10; high-income country).
- “Perhaps there should be also some user/patient layer?” (Expert 10; high-income country).

Unique patient identifier

- “In your figure, is there a master patient registry as a component of the Exchange? If so, I would leave the UPI there, and not repeat it, and all systems in the ecosystem would use that UPI” (Expert 5; LMIC within SSA).

Miscellaneous comments

- “The word ‘levels’ makes me visualise 4 horizontal levels, one above or below the other, rather than the 4 verticals in the diagram. Next, these four ‘levels’ are divided into six ‘sub-layers,’ and I wonder why the word ‘level’ is now replaced by the word ‘layer,’ though my guess is that the ‘sub-layers’ are one hierarchical step below the ‘levels.’ This is a bit confusing when reading it. And once again, the word ‘layers’ makes me imagine six horizontal layers rather than the six vertical ones” (Expert 5; LMIC within SSA).

With respect to the “Governance and Regulation” theme, 17% (2/12) of the experts (expert 7, LMIC outside SSA, and expert 1, Botswana) made 3 suggestions. Expert 7 suggested that this should be the largest theme and contain all other themes, whereas expert 1 suggested the adoption and inclusion of sustainable funding or investment models suited for LMICs. The recommendation of expert 7 was adopted, and the rectangular shape for “Governance and Regulation” was extended to cover all other themes (Figure 1). No change was made regarding funding or investment models as these are already embedded within the “Governance and Regulation” theme.

Regarding the “Interoperability Standards” theme, 25% (3/12) of the experts made 4 suggestions. No changes were made as a result of the suggestion to make the mHeRIF accommodate non–Health Level 7 Fast Healthcare Interoperability Resources standards as the framework already suggests other standards (eg, the International Organization for Standardization and Institute of Electrical and Electronics Engineers 11073 and Digital Imaging and Communications in Medicine). However, suggestions to consider the International Organization for Standardization Technical Report 14639 standard and the inclusion of “IHE Profiles” were adopted and reflected within the mHealth and eRecord standard and the national HIE themes, respectively (Figure 1).

For the “eHealth Software and Infrastructure” theme, 17% (2/12) of the experts made 1 suggestion each. These were to use the term “digital health” as opposed to *eRecords* and differentiate delivery methods, such as on the web and offline, and to have an outer layer in the mobile device interface to independently map telecommunication technologies (eg, SMS text messages, Unstructured Supplementary Service Data, and voice). These did not necessitate any changes to the mHeRIF. The terms “digital health” and *eHealth* are often (incorrectly) used interchangeably; furthermore, the World Health Organization (WHO) considers digital health to be rooted in eHealth, and the latter term is recognized to encompass mHealth and eRecords [3]. Regarding independent mapping of telecommunications technologies, these are already provided for within the “IT Infrastructure” component.

Regarding “Security, privacy and confidentiality,” 8% (1/12) of the experts provided a suggestion. This was to remove “Legislation” as it is already covered under the “Regulation” part of the “Governance and Regulation” component. The premise is debatable—regulations may have no legal standing. Regardless, there will be specific legislation that will affect mHealth and eRecords that must be recognized and considered. To accommodate this issue, a change was made by renaming the “Legislation (Security, Privacy and Confidentiality)” theme (rightmost cross-cutting theme initially) to read “mHealth/eRecords specific legislation (Security, Privacy and Confidentiality)” (Figure 1). For the “Usability” theme, 25% (3/12) of the experts provided 4 suggestions. One suggestion, to include artificial intelligence and human-computer interaction components within the mHeRIF, did not necessitate changes to the mHeRIF. These components are considered aspects under the “Cloud” infrastructure and the “Usability” components within the current framework. Similarly, no changes were made pertaining to the suggestion to include a “user/patient” layer as all users are already provided for within the “mHealth and eRecord stakeholders” component (Figure 1). The suggestion to align the components of the framework with the exemplar architecture did not necessitate any changes as the framework must stand alone. Furthermore, the framework and architecture serve different purposes; hence, the ordering of components within each may require slight variation but does not affect their overall intent. The final suggestion, to further simplify the mHeRIF, did not provide any specific guidance as to what needed to be simplified. However, the framework was reviewed, with no opportunities for simplification identified.

Only 1 suggestion was made regarding the enhancement of the “Unique Patient Identifier” theme. After consideration, no change was made to the mHeRIF. Given that it is almost impossible to have a perfect universal master patient index (MPI), especially within LMICs, it might be more appropriate to enforce the uniqueness of patient records at the individual system level.

A miscellaneous comment was considered overarching and not specific to any theme as it highlighted a general concern over some terminology used within the framework. However, the terms applied were chosen as they were in alignment with terms commonly used in the literature (eg, Refined eHealth European Interoperability Framework). No adjustment of the mHeRIF was considered necessary.

## Discussion

### Principal Findings

Overall, eHealth experts showed general agreement with the structure and components of the mHeRIF (Tables 1 and 2). Similarly, the experts perceived the components of the proposed interoperability architecture derived from the mHeRIF as essential (Table 3). Also worth noting is that experts in Botswana showed greater appreciation and acceptance of the mHeRIF and its components (Tables 1 and 2). However, although the experts from LMICs (within and outside SSA) were generally supportive of the mHeRIF, those from high-income countries were less supportive, citing differing perspectives and expectations. There were only 25% (3/12) of experts who indicated that they would be unable to use the mHeRIF (Table 1). Overall, there was no single framework component that all the experts, or even most, entirely disagreed with, as seen in the responses to the Likert scale questions (Table 1).

Although other interoperability frameworks exist, the overall contribution of the mHeRIF is that it extends the generic and adaptable framework to different country contexts. Similar to the mHeRIF, the OpenHIE specifies mobile devices as an exemplar “point of service application.” However, importantly, the mHeRIF further highlights other considerations (eg, mHealth security and transport standards, data formats and database models, and governance and regulatory considerations) for linking mHealth apps to eRecord systems. These are key for countries seeking to link mHealth apps to other eRecord systems.

As a conceptual framework, the mHeRIF identified core components and themes by arranging them in a logical structure to provide a visual display of how each relates to the others. Therefore, it is essential that the mHeRIF’s components be interpreted in totality and not in isolation. However, the mHeRIF is not prescriptive in terms of what LMICs such as Botswana should do. The first mobile telemedicine initiative to be considered at a national scale in Botswana, the Kgonafalo program [31], was used to demonstrate the utility of the mHeRIF. However, lessons learned from previous eHealth interoperability implementation approaches could further guide the application of the mHeRIF in the context of Botswana. A notable example is from Tanzania, where government leadership buy-in and support were crucial for the successful identification of governance structure for the HIE, including coordination, partnerships, and financing [38]. Consequently, the leadership of the Ministry of Health in Tanzania served as the Chair of the National eHealth Steering Committee, with its information and communications technology unit as the Secretariat and the Project Management Office responsible for coordinating the development of multiple elements of eHealth systems.

In this study, eHealth experts also contributed important considerations for the improvement of the mHeRIF, and these were categorized according to six themes (Textbox 1) as follows: (1) governance and regulation; (2) interoperability standards; (3) eHealth software and infrastructure; (4) security, privacy, and confidentiality; (5) usability; and (6) unique patient identifier (UPI).

The recent WHO Global Strategy on Digital Health (2020-2025) [4] highlights that “digital health can radically change health outcomes if it is supported by sufficient investment in governance.” It further suggests that “actions to strengthening governance should include defining principles and reaching cross-sectoral and international agreements for data sharing, quality and accuracy of health data and prioritization of investment plans and policy.” This could justify one of the findings in this study whereby all eHealth experts (12/12, 100%) either “strongly agreed” or “agreed” that “‘Governance and Regulation’ is a relevant component of the mHeRIF” (Table 1), as well as a suggestion by one of the experts to make it the largest rectangle containing all the other boxes inside (Textbox 1).

Considering that all experts (12/12, 100%) indicated that “fundamental ‘mHealth-eRecord’ standards for interoperability are essential components within the mHeRIF” (Table 1), as is the need to ensure standard compliance, it is also important to consider previously reported challenges to eHealth standardization in LMICs. These include inadequate funding for the standardization process, insufficient human resources, less to no participation in the international standard development process, and inadequate technical infrastructure for standard participation, among others [39].

The WHO Global Strategy [4] further recognizes the use of software global goods, open standards, and common digital health architecture as a means of achieving its strategic objective of promoting global collaboration and advancing the transfer of knowledge on digital health. This could explain some of the study findings that experts agreed to the consideration or use of open-source systems in Botswana. The WHO Digital Health Platform handbook [40] also recognizes the importance of an HIE platform for interoperability and that it should be supported by interoperability standards to enable the bidirectional flow of data and processes among eHealth systems. This aligns with the study finding that all eHealth experts (12/12, 100%) agreed that “the National Health Information Exchange (NHIE) in the mHeRIF is an essential component to linking mHealth solutions to eRecord systems.” Similarly, the need to develop an interoperability framework using the OpenHIE components and standards is also seen as a priority within the Botswana National eHealth Strategy as it is envisaged to facilitate the sharing of information across eRecord systems [15]. The Digital Health Platform handbook [40] further recommends that the HIE should support core services such as authentication, registry, terminology, and reference data and workflow support services. It further highlights that the HIE should interact with external digital health systems using these components (internal functionality) through published standard-based interfaces, such as application programming interfaces and web services.

According to the WHO Digital Health Platform handbook, the HIE should build upon a country’s national eHealth strategy or similar digital health road map and use a requirement-gathering process to determine which applications and components are needed to realize national objectives [40]. This further highlights that HIE needs should be planned and designed using architectural methods. These should show how different components fit together and interact (eg, an enterprise architecture to describe how the HIE components will interact with each other and external systems). In this study, an architectural model was derived from the mHeRIF and perceived by most experts (10/12, 83%) to have fundamental concepts and themes to support linking the mobile telemedicine program (Kgonafalo) in Botswana with other eRecord systems (Table 3).

The need for a UPI was highlighted by most experts (9/12, 75%; Table 1), with 8% (1/12) further suggesting having the UPI under the master patient registry (Textbox 1). Although having a universal UPI is the goal, it is seldom achieved, and challenges have been experienced, leading to varying MPI implementation approaches [41-43]. Jayatissa [44] also highlighted that the MPI should “be self-contained so that it can easily be implemented in any health facility; be able to adapt to existing systems, not against the other; use all standards defined and accepted by the community in order to achieve, communicate with all entities; and comply with all basic functions since no system is successful if it is incomplete.” A previous study by Sragow et al [45] further suggests that “comprehensive patient identification could accurately and efficiently integrate fragmented patient data to create a more complete record while mitigating the incorrect linkage of health care data belonging to other patients.”

Security, privacy, and confidentiality issues were considered important components of the mHeRIF by all eHealth experts in this study (12/12, 100%; Table 1). These were also previously raised, highlighting key issues such as who has the right to access health care records, either patients or health care professionals, and ensuring that only approved applications or devices will be able to access sensitive health care data [46]. Similarly, the need to “comply with data protection requirements and ethical guidelines (e.g., regarding privacy) that impact on the processing of healthcare data” was also emphasized [44], as was the need to align with the Botswana Data Protection Act [47] within the proposed mHeRIF. Although suggested within the mHeRIF, and considering the benefits of cloud computing technologies such as efficient processing and data storage with less expense to end users, several data security challenges (data transfer and storage issues) in both mHealth devices and cloud storage have been previously identified, as well as the need for a comprehensive information security framework for mHealth services [46].

Several comments were made pointing to “usability” of the mHeRIF. An expert even suggested the inclusion of a *user/patient layer* within the mHeRIF (expert 10; high-income country). However, the authors considered this to be provided for within the “mHealth systems and stakeholders” and “eRecord systems and stakeholder” components of the mHeRIF. Furthermore, these groups could pursue interoperability under each of the 4 levels: legal, organizational, semantic, and technical or syntactic.

A few negative responses were also noted, mostly related to the experts’ lack of knowledge of Botswana-specific policy documents. There were also differing interpretations of the framework’s intent. For example, an expert from an LMIC within SSA expected the mHeRIF to function as an interoperability model versus a framework. However, the terms “model” and “framework” are not considered synonymous. Both are simplified visual tools intended to describe but not explain. However, a “model” portrays something more definitive (often a physical representation) to be used as an example to follow or imitate closely and that simplifies the *process* of translating research into practice [48]. In contrast, a “framework” is more of a basic conceptual structure intended to offer a “high-level” overview that identifies factors believed to influence an outcome. The latter, it is believed, will be appreciated by and helpful to policy makers and others. Thus, the mHeRIF, as intended, grows awareness of issues but does not provide a precise and detailed “how to” guide. In one case (expert 5; LMIC within SSA), this type of misunderstanding affected subsequent ratings as the expert felt that they could not comfortably respond to several further survey questions until their first query had been addressed.

Interestingly, some seeming contradictions identified a potential issue. An expert (expert 7) from an LMIC outside SSA gave a “No” response to question 12 (“The mHeRIF (Figure 1) shows the need to review and align ‘mHealth-eRecord collaboration agreements’ and ‘mHealth-eRecord workflow agreements’?”; Table 2) yet responded “Agree” to the similar statement from the previous “framework design” section (question 21: “The need to review and align ‘mHealth-eRecord collaboration agreements’ and ‘mHealth-eRecord workflow agreements’ within the mHeRIF is essential”; Table 1). Similarly, expert 10 (LMIC) responded “No” to question 4 (Table 2) but responded “Strongly Agree” to statement 13 (Table 1), as well as “No” to question 6 (Table 2) but “Strongly Agree” to statement 15 (Table 1). Collectively, these apparent contradictions suggested a flaw in the mHeRIF when considering the sense of these questions versus the statements; although each expert agreed with the “need,” for them, the “need” was not adequately reflected in the mHeRIF. This was debated, but no means of demonstrating “need” more clearly was felt feasible.

The mHeRIF was considered essential by most eHealth experts in this study (9/12, 75%; Table 1). The application of the mHeRIF may require additional resources (financial, human, and technical) to set up, implement, and sustain it. The Ministry of Health in Botswana is currently working toward implementing interoperable eHealth solutions across the health sector as guided by the National eHealth Strategy. The mHeRIF is currently under consideration as the framework for mHealth and eRecord interoperability.

### Limitations

There were few eHealth experts to choose from with the requisite knowledge of interoperability concepts. Furthermore, the mHeRIF does not provide finer technical details for implementation but, rather, offers guidance on key considerations for linking mHealth apps to eRecord systems. This was considered a limitation by an eHealth expert from a high-income country but is in alignment with the concept of a “framework.” Finally, there is no national experience of linking mHealth apps to eRecord systems in Botswana, necessitating empirical testing of the mHeRIF.

### Conclusions

The mHeRIF provides an opportunity to enhance the Botswana National eHealth Strategy by informing the linking of mHealth apps to existing eRecord systems, an essential component of the “Standards and Interoperability” pillar within the strategy. The framework will inform implementation approaches while considering all essential components, standards, and varying infrastructure needs, hence reducing the failure rates of most mHealth and eRecord initiatives in Botswana. Given the absence of a national framework for linking mHealth apps to the eRecord system in Botswana, this gap has been filled by introducing the mHeRIF—which is intentionally generic in its design. Considering its generic nature, the mHeRIF may require adaptation to suit varying use-case scenarios within Botswana and elsewhere. The mHeRIF and its exemplar architecture model were validated by eHealth experts, and their suitability in the context of Botswana was confirmed. The study findings offered valuable insights and suggestions for enhancing the mHeRIF. Further studies could confirm its efficacy in ensuring the linking of mHealth apps to eRecord systems as the mHeRIF is applied in Botswana.

## Appendix

Multimedia Appendix 1 National eHealth Strategy, digital health platform architecture published by the International Telecommunication Union.

Multimedia Appendix 2 Original Mobile Health–Electronic Record Interoperability Framework.

Multimedia Appendix 3 mHeRIF Explanatory Notes.

## Abbreviations

EHR: electronic health record
eRecord: electronic record
HIE: health information exchange
LMIC: low- and middle-income country
mHealth: mobile health
mHeRIF: Mobile Health–Electronic Record Interoperability Framework
MPI: master patient index
OpenHIE: Open Health Information Exchange
REDCap: Research Electronic Data Capture
SSA: sub-Saharan Africa
UPI: unique patient identifier
WHO: World Health Organization

## Data Availability

The data collected and analyzed during this study are available from the corresponding author upon reasonable request.
