# Supplementary figures and images for: Validation of an Interoperability Framework for Linking mHealth Apps to Electronic Record Systems in Botswana: Expert Survey Study
Source: JMIR Form Res. 2023 May 2;7:e41225. doi: 10.2196/41225 (PMC10189626; doi:10.2196/41225)

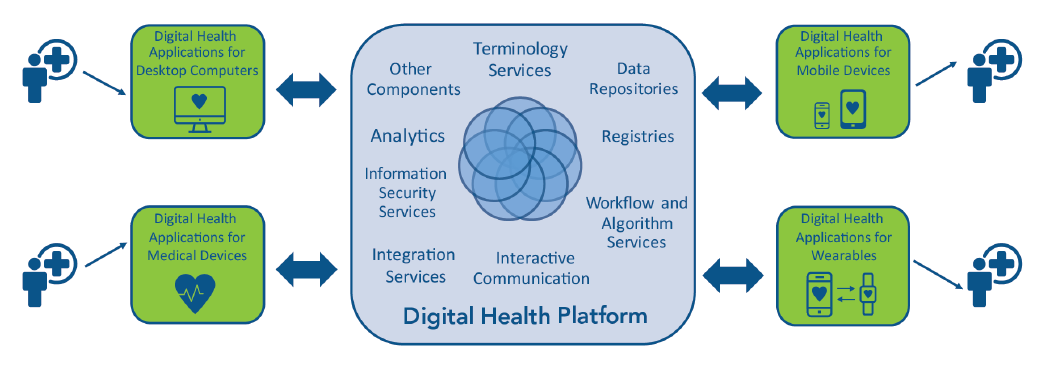

Supplement: Multimedia Appendix 1 [file formative_v7i1e41225_app1.png]

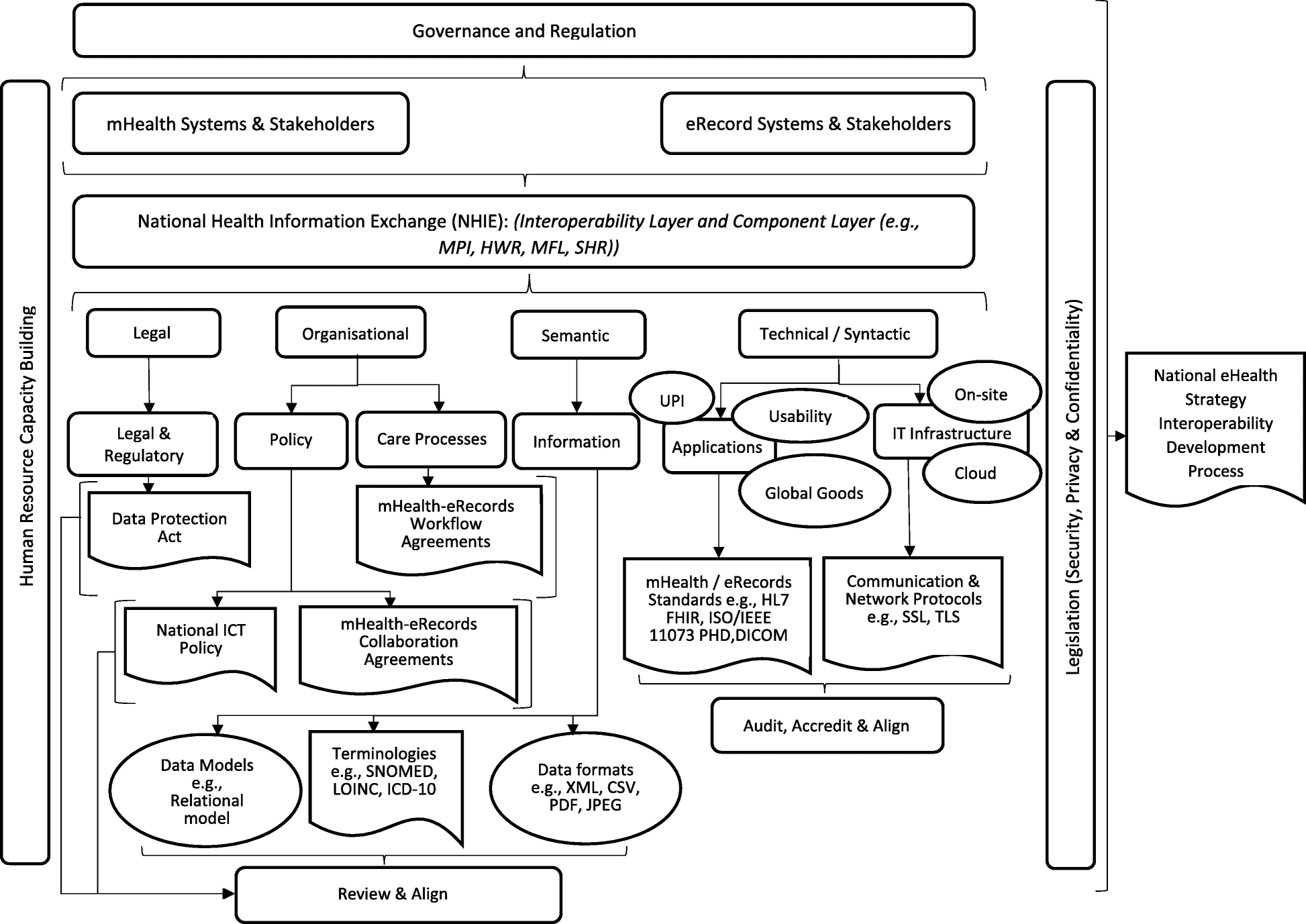

Supplement: Multimedia Appendix 2 [file formative_v7i1e41225_app2.png]
