# Supplementary material for: Validation of an Interoperability Framework for Linking mHealth Apps to Electronic Record Systems in Botswana: Expert Survey Study
Source: JMIR Form Res. 2023 May 2;7:e41225. doi: 10.2196/41225 (PMC10189626; doi:10.2196/41225)
Supplement: Multimedia Appendix 3 [file formative_v7i1e41225_app3.pdf]

Explanatory notes for the proposed mHealth-eRecords Interoperability Framework (mHeRIF) for Botswana and similar developing countries.

My name is Kagiso Ndlovu, and I am a PhD (Telehealth) Research Student at the University of KwaZulu–Natal, Durban, South-Africa. My research has involved consideration of two major components of ehealth – mHealth and eRecords – in particular the linking of these components together, something poorly addressed in the literature and not at all in Botswana's National eHealth strategy. As part of my PhD research, I have developed an mHealth-eRecord Interoperability Framework (mHeRIF) intended for use in Botswana and other similar developing countries. The framework's conceptualisation and design was guided by literature findings, including consultations with eHealth experts, and existing frameworks. These collectively identified key elements, concepts, and standards relevant and essential for inclusion [16, 28]. Specific guidance was derived from established frameworks, such as: 1. the OpenHIE framework (a mission-driven Community of Practice including countries, organisations, individuals and donors working to promote sharing of health data across many different software products) [30] ; 2. Botswana's National eHealth Strategy (recommendations for use of open-source frameworks and tools that will be compatible and align with the strategy) [15] ; and 3. the Refined eHealth European Interoperability Framework (ReEIF) which splits two of the original interoperability levels (Organisational and Technical) into two sub-levels each (Organisational: Policy and Care Process; Technical: Applications and IT Infrastructure), yielding six levels) [27]. The overall aim of the study is to have a framework suitable for use in developing countries and that could be used to guide greater interoperability between these components, allowing their individual and combined contributions in the future to be maximised.

mHealth / eRecord interoperability has been defined as “The ability of two or more systems to exchange information and use the information that has been exchanged” [16, 28].

As an expert, you are requested to contribute to this study by assessing the suitability for use of the developed mHeRIF (presented below) by reviewing the construct and content of the framework and providing your expert opinion. Taking part in this study is completely voluntary. You have the right to not participate and the right to withdraw from the study at any time without any consequences.

Instructions for Participants

If you are willing to participate, you are requested to first review Figure 1 and 2 and their accompanying explanations (this should take about 20 minutes) before proceeding to respond to the questionnaire (this should take about 15-20 minutes). Your views will help confirm or refute the need for any of these elements to be considered or modified in the final mHeRIF intended for use by Botswana and other similar developing countries.

mHeRIF description

At the top of Figure 1, the framework first illustrates the overarching need for mHealth and eRecord systems governance and regulation which in turn impacts mHealth and eRecords systems and stakeholder coordination, collaboration, compliance with national policies and standards defined within the national health information exchange (NHIE). The framework then illustrates that interoperability will ideally be attained across four distinct levels (Legal, Organisational, Semantic, and Technical/Syntactic). According to the ReEIF these would be

further refined into the six sub-layers seen in the next level of the diagram (Legal & Regulation, Policy, Care Processes, Information, Applications, and IT Infrastructure). Themes, concepts, elements and standards identified from prior studies [16, 28] informed specific details about each interoperability sub-layer. For example, the 'Applications' sub layer accommodated the aspects 'Usability', 'Unique Patient Identifier (UPI)', and the 'Global goods' concept. Similarly, the 'IT Infrastructure' sub-layer accommodated concepts such as 'Cloud' and 'On-site' server infrastructures. Other concepts aligned to appropriate sub-layers included the Botswana 'Data Protection Act' (DPA), 'mHealth-eRecord Workflow Agreements', 'mHealth-eRecord Collaboration Agreements','Terminologies' (e.g. SNOMED-CT, LOINC, ICD-10), 'Data Models' (e.g. Relational Data Model), and 'Data formats' (e.g. XML, JSON, CSV). Standards, under 'Applications', included HL7-FHIR, ISO/IEEE 11073 (PHD), DICOM, while standards under 'IT Infrastructure' included Secure Socket Layer (SSL) encryption or Transport Layer Security (TLS) standards. All of these will require regular review, accreditation and alignment to the National eHealth Strategy Interoperability Development Process (far right-hand side of the diagram).

The framework also highlights cross-cutting themes (Figure 1). These include the 'Human Resource Capacity Building' (left-hand side of the diagram) and all legislation impacting 'Security, Privacy and Confidentiality' concerns (right-hand side of the diagram). All of these feed into a comprehensive and informed 'National eHealth Strategy Interoperability Development Process' (far right-hand side of the diagram) supporting linking of mHealth solutions to eRecord systems. This would align with the Botswana Interoperability Pillar outlined in the National eHealth Strategy.

Proposed Architecture description

Although a generic framework, the proposed mHeRIF may require modest adaptation below the 'legal, organisational, semantic, and technical' sub-layer that will allow the enterprise architecture to better suit each specific implementation. To demonstrate this, the framework's functionality was used to propose an OpenHIE compliant architecture for linking the Kgonafalo mobile telemedicine programme to eRecord systems in Botswana (Figure 2). The mHealth solution and eRecord system are identified as Point of Service Applications (POSA), linking directly or indirectly with the HIE. The Mobile Device Translation Layer FIHR Interface supports implementation of various mobile devices and platforms (e.g., iOS, Microsoft, Android). Here, the Kgonafalo mobile solution would be linked to an EMR system through the interoperability layer supporting specific services including the 'Case Notification Service (CNS)'. The CNS would be responsible for sending bi-directional medical case notifications across mHealth and eRecord systems, for example, when a new case is registered using the mHealth solution and resolved through the eRecord system (e.g., an EMR). Various repositories (e.g., Master Patient Index (MPI), Master Facility List (MFL), Shared Health Record (SHR)) all work within the OpenHIE framework [30]. The architecture would have the DHIS2 platform as the main repository containing aggregate level content from the various registries. The Integrating the Healthcare Enterprise (IHE) workflow profiles (endorsed by the European Commission [32]) would support the various healthcare scenarios for the Kgonafalo mobile telemedicine programme. The Exchange of Personal Health Record Content (XPHR) integration profile would describe the content and format of summary information extracted from a PHR System for import into an EHR System, and vice versa.

The Cross-Enterprise Document Sharing Medical Summaries (XDS MS) profile would describe content and format of discharge summaries and referral notes. Unique Patient Identification across systems would be supported by the Patient Identifier Cross-referencing (PIX) and the Patient Demographics Query (PDQ) Integration Profile. A Scheduled Workflow (SWF) would integrate ordering, scheduling, imaging acquisition, storage and viewing for examinations. The Audit Trail and Node Authentication (ATNA) Integration Profile would support basic security through functional access controls, defined security audit logging and secure network communications.

Specific interoperability standards supporting the proposed IHE profiles would be embedded within the interoperability layer (OpenHIM). The 'Mediator' service of the HIE would handle queries and responses between different database systems and resolve complex orchestration of communications between multiple mHealth solutions and eRecord systems. As noted in the methods, the mHeRIF may require modest adaptation for specific services. In Figure 2, examples are shown within broken line boxes where alternate considerations have been incorporated within this proposed architectural solution for the Kgonafalo mobile telemedicine programme to illustrate this. These were use of an unstructured supplementary service data (USSD) option and the short message service (SMS). Lastly, security and audit services are essential (left-hand side of the diagram) and encompass all of the architecture components. This example demonstrates flexibility in the application of the mHeRIF.

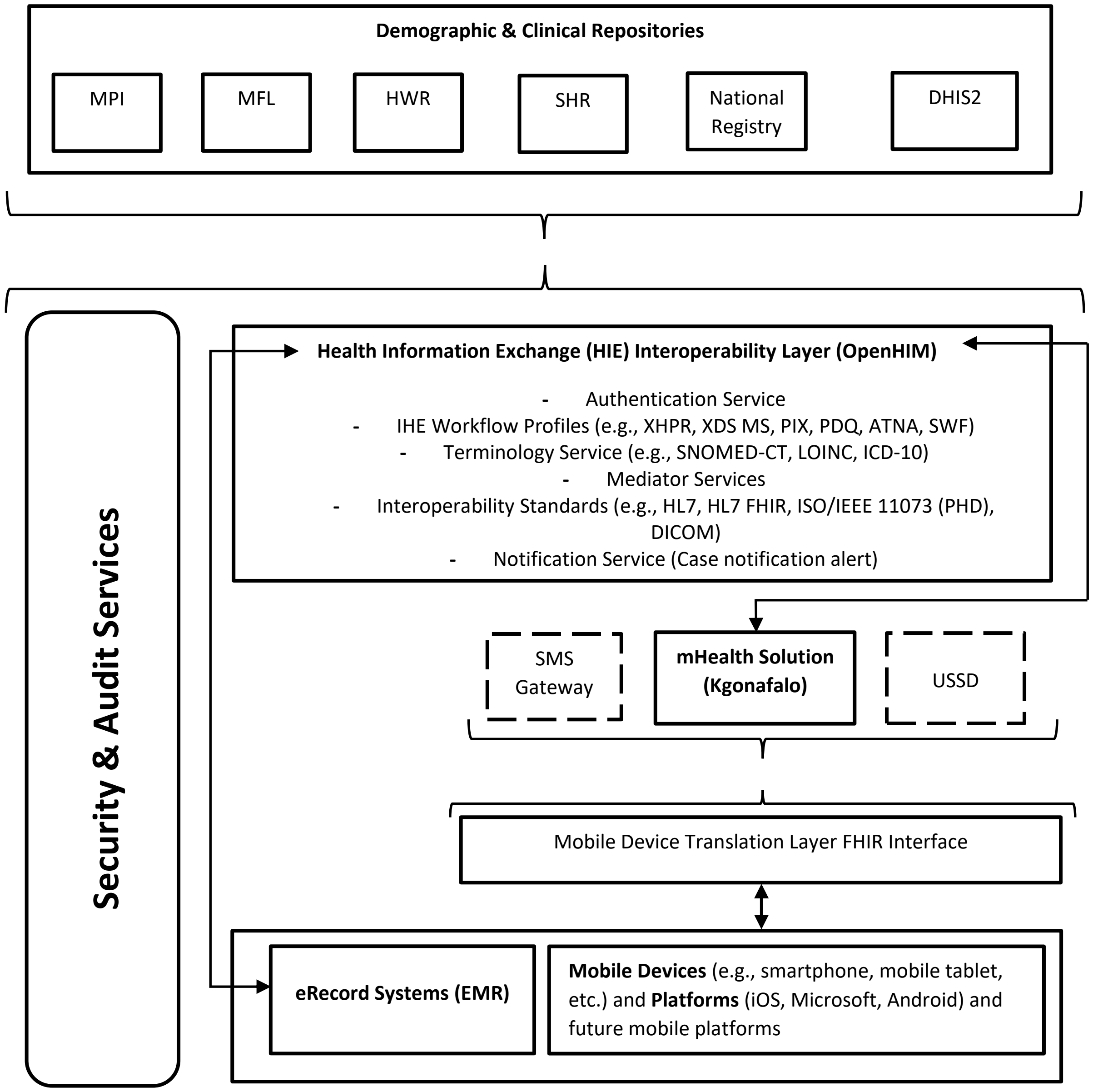

Figure 2. Proposed interoperability architecture design for Kgonafalo mobile telemedicine programme using mHeRIF.

Abbreviations / acronyms: **ATNA:** Audit Trail and Node Authentication; **DICOM:** Digital Imaging and Communications in Medicine; **DHIS2:** District Health Information System version 2; **FHIR:** Fast Healthcare Interoperability Resources; **HWR:** Health Worker Registry; **HL7:** Health Level 7; **ICD-10:** International Classification of Disease – 10; **IHE:** Integrating the Healthcare Enterprise; **ISO/IEEE 11073 (PHD):** International Standards Organisation/Institute of Electrical and Electronics Engineers 11073 (Personal Health Data); **LOINC:** Logical Observation Identifiers Names and Codes; **MFL:** Master Facility List; **MPI:** Master Patient Index; **PDQ:** Patient Demographics Query; **PIX:** Patient Identifier Cross-referencing; **SHR:** Shared Health Record; ; **SMS:** Short Message Service; **SNOMED-CT:** Systematized Nomenclature of Medicine - Clinical Terminologies; **SWF:** Scheduled Workflow; **USSD:** Unstructured Supplementary Service Data; **XDS MS:** Cross-Enterprise Document Sharing Medical Summaries; **XPHR:** Exchange of Personal Health Record
